# The Effects of Different Zn Forms on Sintering Basic Characteristics of Iron Ore

**DOI:** 10.3390/ma17122919

**Published:** 2024-06-14

**Authors:** Jiantao Ju, Jian Zu, Xiangdong Xing, Lei Yang, Xinru Xiang

**Affiliations:** 1 School of Metallurgical Engineering, Xi’an University of Architecture and Technology, Xi’an 710055, China; ju_jiantao@163.com (J.J.); 15309216845@163.com (J.Z.); 15795082077@163.com (X.X.); zqm980513@163.com (X.X.); 2 Research Center of Metallurgical Engineering Technology of Shaanxi Province, Xi’an 710055, China

**Keywords:** zinc, sintering basic characteristics, first-principles calculations, thermodynamic calculation

## Abstract

The micro-sintering method was used to determine the sintering basic characteristics of iron ore with Zn contents from 0 to 4%, the influence mechanism of Zn on sintering basic characteristics of iron ore was clarified by means of thermodynamic analysis and first-principles calculations. The results showed that (1) increasing the ZnO and ZnFe_2_O_4_ content increased the lowest assimilation temperature (LAT) but decreased the index of liquid phase fluidity (ILF) of iron ore. The addition of ZnS had no obvious effect on LAT but increased the LIF of iron ore. (2) ZnO and ZnFe_2_O_4_ reacted with Fe_2_O_3_ and CaO, respectively, during sintering, which inhibited the formation of silico-ferrite of calcium and aluminum (SFCA). The addition of ZnS accelerated the decomposition of Fe_2_O_3_ in the N_2_ atmosphere; however, the high decomposition temperature limited the oxidation of ZnS, so the presence of ZnS had a slight inhibitory effect on the formation of SFCA. (3) The Zn concentrated in hematite or silicate and less distributed in SFCA and magnetite in the form of solid solution; meanwhile, the microhardness of the mineral phase decreased with the increase in Zn-containing solid solution content. As the adsorption of Zn on the SFCA crystal surface was more stable, the microhardness of SFCA decreased more. The decrease in microhardness and content of the SFCA bonding phase resulted in a decrease in the compressive strength of the sinter.

## 1. Introduction

At present, the main iron-bearing charge of blast furnaces in China remains the sinter, accounting for about 70–80% of the blast furnace burden [1,2,3]. Hence, the chemical composition of the sinter significantly affects the operation stability of the blast furnace. Zn compounds in sinter are reduced to Zn vapor in the high-temperature area of the blast furnace, most of Zn vapor is oxidized to ZnO, which is adsorbed on the surfaces of charges and furnace wall, resulting in the worsening of permeability of the charges and corrosion of refractories [4,5,6,7]. In recent years, domestic steel enterprises have regarded metallurgical dust generated in the steel production process as sintering raw materials to control the cost and reduce the pollution [8,9]. Metallurgical dust not only contains abundant Fe and C resources but also contains large amounts of harmful elements such as Zn [10,11,12]. The recycling of metallurgical dust by sintering increases the Zn content in the sinter. Therefore, it is necessary to reveal the effect of Zn on sintering basic characteristics of iron ore, which provides theoretical guidance for optimizing sintering parameters to produce high-quality sinter.

The forms of Zn in sintering raw materials are various. Zn mainly presents in the form of sphalerite (ZnS) and zincite (ZnO) in natural ores [13]. Wang et al. [14] adopted X-ray absorption spectroscopy (XAS) to examine the Zn-containing phase in metallurgical dust, and the results showed that Zn mainly existed in the form of ZnO and ZnFe_2_O_4_ in metallurgical dust. In the last few years, previous studies have mainly focused on the sintering behavior of Zn compounds. Wang et al. [15] investigated the phase transformation of Zn during sintering by thermodynamic calculation, and the results identified that the Zn compounds such as ZnS, ZnO, and Zn_2_SiO_4_ in industry sintering blends were mostly transformed into ZnFe_2_O_4_, only ZnCl_2_ and a minimal amount of Zn reduced from ZnO were removed by vaporization during sintering. Lv et al. [16] studied the reaction behavior of ZnO under the reducing atmosphere, and the results showed that increasing the temperature and decreasing the CO content were conducive to the conversion of Fe^2+^ to Fe^3+^, which correspondingly restrained the reduction of ZnO. Although revealing the sintering behavior of Zn compounds contributes to improving the removal rate of Zn during sintering, there is a threshold for the Zn removal rate. Over the last few decades, many metallurgists have begun to study the effect of gangue on the sintering basic characteristics of iron ore [17,18,19,20], nevertheless, there is little research on the effect of Zn on the sintering basic characteristics of iron ore, and relevant studies only stay at the level of pure reagent [21]. The mechanism that influences the high-temperature sintering behavior of Zn compounds on the sintering basic characteristics of iron ore is unclear.

As mentioned above, Zn in sintering raw materials is mainly in the form of ZnO, ZnFe_2_O_4_, and ZnS. In this paper, the chemical reagents ZnO, ZnFe_2_O_4_, and ZnS were chosen as Zn resources to constitute the experimental samples with iron ore. Thermogravimetry (TG) was performed on the samples of iron ore containing different contents and forms of Zn to investigate the thermal decomposition behavior during sintering. The sintering basic characteristics of iron ore were measured by the micro-sintering method; meanwhile, first-principles calculations and thermodynamic calculations were used to clarify the influence mechanism of Zn on the sintering basic characteristics of iron ore.

## 2. Materials and Methods

### 2.1. Materials

**Figure 1:**
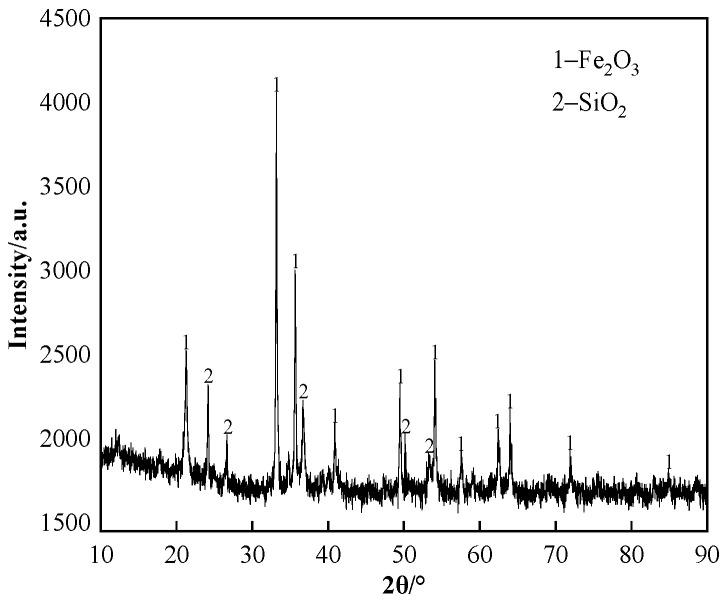
The XRD pattern of iron ore.

**Figure 2:**
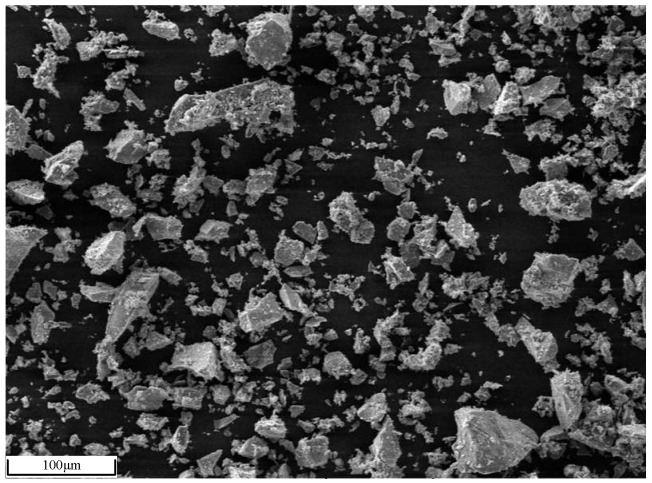
The SEM photo of iron ore.

The chemical composition of iron ore provided by a domestic sintering plant is shown in Table 1, and the ZnO content in iron ore is no more than 0.01%. The X-ray diffraction (XRD) pattern and Scanning electron microscope (SEM) photo of iron ore are shown in Figure 1 and Figure 2, respectively. Figure 1 indicates that the main iron-bearing mineral is hematite. Before preparing the experimental sample, iron ore and chemical reagents were ground to powder with a particle size of less than 0.15 mm and heated in the oven at 100 °C for 3 h to eliminate the effects of particle size and moisture, respectively.

The chemical composition of iron ore with different contents and forms of Zn is shown in Table 2. The Zn content in the iron ore was limited to 0–4%; ZO, ZF, and ZS represent ZnO (≥99.0%, XiLong Scientific, Chengdu, China), ZnFe_2_O_4_ (≥99.0%, LeYan, Shanghai, China), and ZnS (≥99.0%, Rhawn, Shanghai, China), respectively.

### 2.2. Methods

#### 2.2.1. Micro-Sintering Experiment

Referring to relevant literature [22,23], the micro-sintering experiment is as follows:

1. Assimilation performance

**Figure 3:**
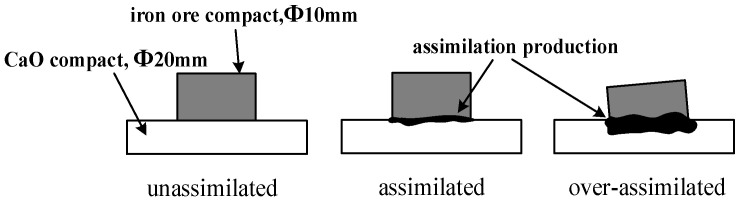
The schematic diagram of the assimilation process.

Based on the data in Table 2, the ore powder and chemical reagents were thoroughly mixed with an appropriate amount of anhydrous ethanol for 30 min and then dried in the electrothermal blowing dry box (101-0ASB, ±1 °C, Beijing Kewei Yongxing Instrument Co., Ltd., Beijing, China) at 100 °C for 2 h. The experimental sample was obtained by milling the dried mixture in a ball milling for 20 min. The 1.0 g experimental sample and 2.5 g CaO (≥98.0%, Damao, Tianjin, China) were weighed and filled into a steel mold and then pressed into iron ore compact with a diameter of 10 mm and CaO compact with a diameter of 20 mm under 20 MPa, respectively. The iron ore compact was placed on the CaO compact and then sent into the micro-sintering device (BR-14AS-12, 1400 °C, Zhengzhou Bona Heat Kiln Co., Ltd., Zhengzhou, China) for roasting. The temperature was first raised to 1000 °C and then continued to rise at a heating rate of 3 °C/s until the assimilation characteristic appeared at the contact interface between the iron ore compact and CaO compact; the corresponding temperature was defined as the lowest assimilation temperature (LAT). During the heating stage, N_2_ with 1 L/min was continuously injected into the micro-sintering device to promote the decomposition of Fe_2_O_3_ to form Fe_3_O_4_, which was basically equivalent to the sum of the reduction and decomposition reaction of Fe_2_O_3_ in the actual sintering process. The assimilation process is shown in Figure 3.

2. Liquid Phase Fluidity

**Figure 4:**
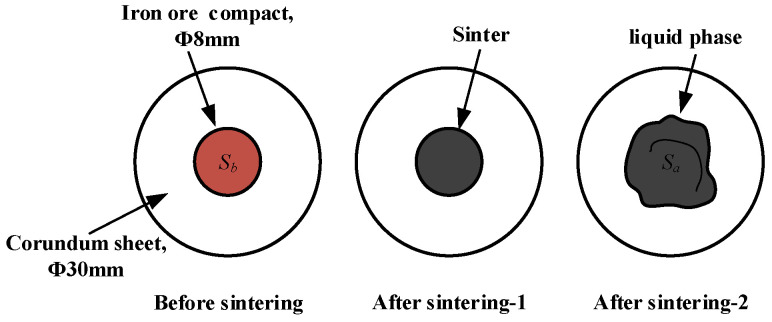
The schematic diagram of the liquid phase flow process.

The ore powder and Zn compounds were configured into the experimental sample with a basicity of 4.0 by CaO, and the 0.8 g experimental sample was weighed and filled into a steel mold and then pressed into iron ore compact with a size of Φ8 × 5 mm. The iron ore compact was placed on the corundum sheet with a diameter of 30 mm and then sent into the micro-sintering device for roasting. The temperature was raised to 1280 °C and then held for 4 min. During the sintering process, N_2_ with 1 L/min (heating and holding stage) and O_2_ with 0.5 L/min (cooling stage) were continuously injected. When the internal temperature of the device dropped to room temperature, the corundum sheet was taken out to calculate the index of liquid phase fluidity (ILF). The liquid phase flow process is shown in Figure 4, and the calculation of ILF is shown in Equation (1).

ILF = (S_a_ − S_b_)/S_b_ (1)

3. Silico-Ferrite of Calcium and Aluminum (SFCA) Formation Characteristics

The ore powder and Zn compounds were configured into the experimental sample with a basicity of 2.0 by CaO, and the 2.0 g experimental sample was weighed and filled into a steel mold and then pressed into iron ore compact with a size of Φ15 × 8 mm. The iron ore compacts were placed into a corundum crucible with a volume of 20 mL and then sent into the micro-sintering device for sintering. The experimental temperature regime and atmosphere control were the same as the liquid phase fluidity experiment. When the internal temperature of the device dropped to room temperature, the corundum crucible was taken out. The sintered compacts were analyzed by X-ray diffraction instrument (D8 Advance A25, Bruker AXS, Karlsruhe, Germany) with copper target, which was operated at 40 kV and 40 mA in step mode with 0.01°2θ step and a count time of 0.15 s per step over a 2θ range from 10° to 90°, to determine the mineral phase composition and investigated by Scanning electron microscope–Energy dispersive spectroscopy instrument (Gemini SEM 300, Carl Zeiss AG, Oberkochen, Germany) to observe the microstructure and determine the relative content of mineral phase.

4. Compressive strength

The experimental procedures were the same as those used in the SFCA formation characteristic experiment. The compressive strength of the sintered compact was measured by an electronic pressure tester (LD-YB-2, ±1 N, Wuzhou Special Equipment Factory, Wuzhou, China).

#### 2.2.2. Microhardness Detection

The microhardness of the mineral phase in the sintered compact obtained by the compressive strength experiment was measured by a Semi-automatic micro-Vickers hardness tester (401 MAD, ±2 HV, JVC, Yokohama, Japan). Before measuring the microhardness of the mineral phase, the sintered compact was embedded in a rubber mold with a size of Φ24 × 12 mm by a mixture of epoxy resin curing agent and acrylic powder and then disposed by abrasive paper and a Semi-automatic polishing machine (XP 810E, Guangzhou Jingying Chemical Technology Co., Ltd., Guangzhou, China). The microhardness of the mineral phase was the average sum of the hardness values at 10 different points on the mineral phase surface. The Vickers hardness value (HV) calculation is shown in Equation (2) [24]. F and d represent a fixed experimental load (100 g) and the arithmetic average of the diagonal of the diamond indentation, respectively.

HV = 0.1891(F/d^2^) (2)

#### 2.2.3. TG Experiment

The experimental samples configured in the compressive strength experiment were used in the Thermogravimetry (TG) experiment. The 50 mg experimental sample was filled into the corundum crucible and then placed into the synchronous thermal analyzer (STA449F3, Netzsch Instrument Inc., Selb, Germany). The temperature regime was set as room temperature—1350 °C, non-isothermal heating. Before heating up, O_2_ at 20 mL/min and N_2_ at 50 mL/min was injected to discharge the impurity gas, and then N_2_ protection gas at 20 mL/min was injected during the heating stage.

#### 2.2.4. Thermodynamic Calculation

The reaction thermodynamics of Zn compounds in this paper were investigated as follows: Firstly, primary compounds in the experimental samples and reaction equations were listed. Secondly, the reaction conditions were confirmed. Thirdly, the thermodynamic properties and reactions of Zn compounds were calculated by the “reaction” module in FactSage 7.1. Finally, the properties of the sintering equilibrium liquid phase of experimental samples containing different contents and forms of Zn were calculated by “Equilib” and “Viscosity” modules.

## 3. Results and Discussion

**Figure 5:**
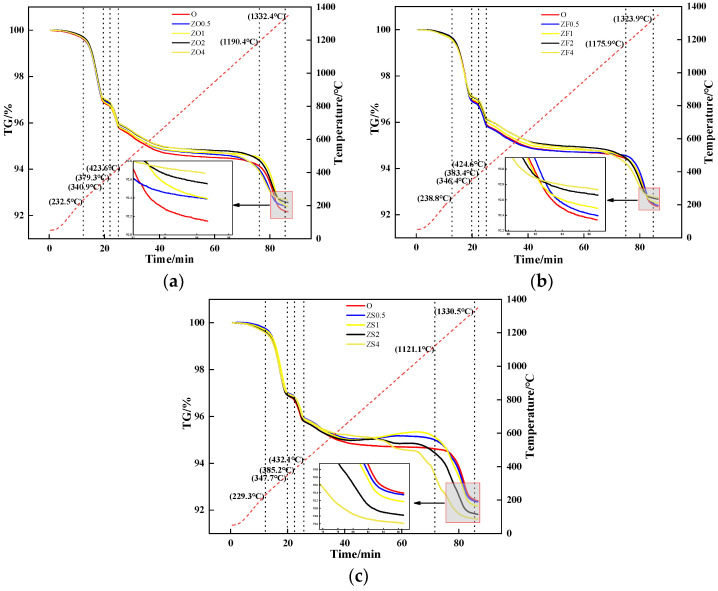
TG–time relationship of experimental samples containing different contents and forms of Zn: (**a**) ZnO; (**b**) ZnFe_2_O_4_; (**c**) ZnS.

The TG–time relationship of experimental samples (R = 2.0) containing different contents and forms of Zn is shown in Figure 5. The experimental sample without Zn started to have a mass loss at about 250 °C, 380 °C, and 1200 °C during the heating process, which corresponded to the decomposition of crystalline water, Ca(OH)_2_, and hematite (Fe_2_O_3_), respectively. When Zn content increased from 0 to 4%, Figure 5a,b shows that the TG curve increased from 92.19% to 92.69% (ZnO) and 92.77% (ZnFe_2_O_4_) at the end of the Fe_2_O_3_ decomposition stage, respectively; hence, ZnO and ZnFe_2_O_4_ inhibited the decomposition of Fe_2_O_3_. Figure 5c shows that the TG curve decreased from 92.19% to 91.61% (ZnS) at the end of the Fe_2_O_3_ decomposition stage; hence, ZnS promoted the decomposition of Fe_2_O_3_.

### 3.1. The Effects of Different Forms of Zn on Assimilation Performance

**Figure 6:**
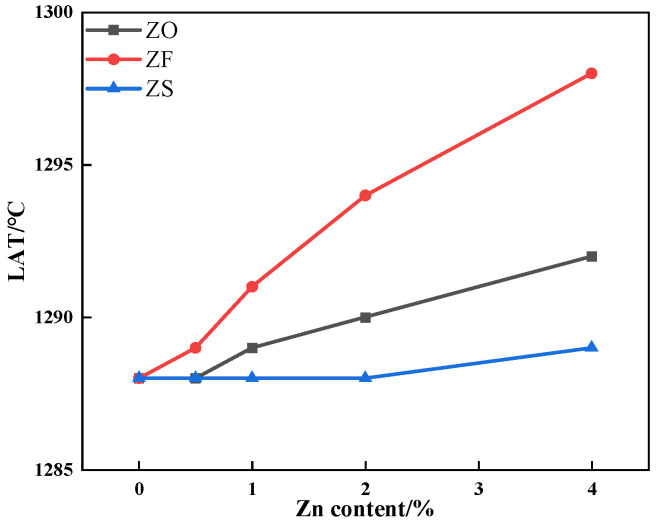
The effects of different forms of Zn on the LAT of iron ore.

The results of the assimilation experiment of iron ore containing different contents and forms of Zn are shown in Figure 6. As can be seen from Figure 6, the lowest assimilation temperature (LAT) of iron ore without Zn is 1288 °C, which belongs to medium assimilation performance. With increasing the Zn content, the LAT of iron ore containing ZnO and ZnFe_2_O_4_ increased, while that of iron ore containing ZnS did not change significantly. When the Zn content increased from 0 to 4%, the LAT of iron ore containing ZnFe_2_O_4_ increased the most, from 1288 °C to 1298 °C.

**Figure 7:**
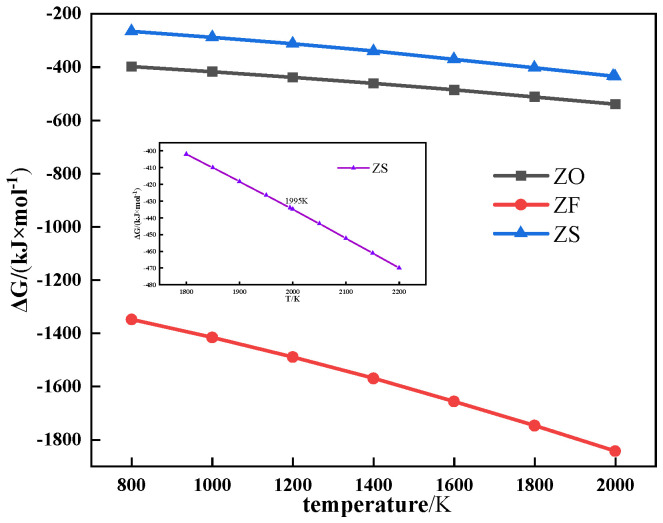
The relationship between Gibbs free energy and temperature of different forms of Zn.

Figure 7 shows the thermochemical properties of ZnO, ZnFe_2_O_4_, and ZnS. As shown in Figure 7, ZnO, ZnFe_2_O_4_, and ZnS were all refractory in the sintering process.

**Figure 8:**
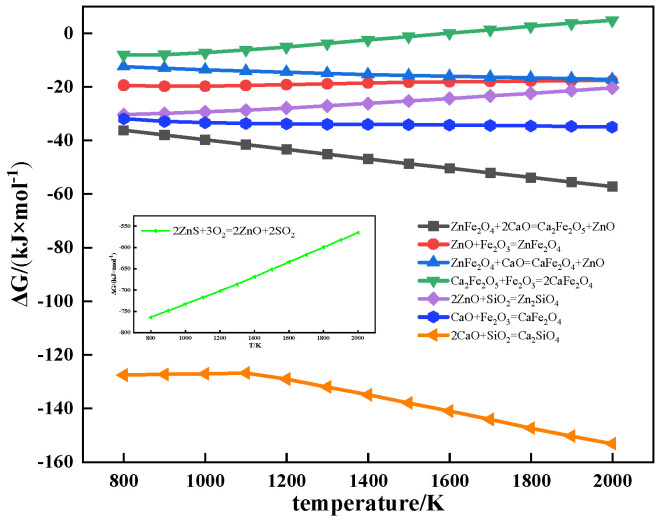
The relationship between Gibbs free energy and temperature of partial reactions during sintering.

The relationship between Gibbs free energy and temperature of partial reactions during sintering is shown in Figure 8. ZnO could react with Fe_2_O_3_ to form ZnFe_2_O_4_ during sintering, which restrained the formation of low-melting calcium ferrite (CaFe_2_O_4_, 1216 °C), and the LAT of iron ore increased. However, the ΔG of the ZnFe_2_O_4_ formation reaction was higher than that of the CaFe_2_O_4_ formation reaction. Therefore, the addition of ZnO had limited influence on the formation of CaFe_2_O_4_ during sintering. The high-melting dicalcium ferrite (Ca_2_Fe_2_O_5_, 1449 °C) was formed by a solid-phase reaction between ZnFe_2_O_4_ and CaO during sintering. The ΔG of the Ca_2_Fe_2_O_5_ formation reaction was lower than that of the CaFe_2_O_4_ formation reaction. Therefore, the addition of ZnFe_2_O_4_ had a significant influence on the formation of CaFe_2_O_4_ during sintering. In the sintering process, the O_2_ produced by the decomposition of Fe_2_O_3_ oxidized ZnS to ZnO. However, the decomposition temperature of Fe_2_O_3_ limited the oxidation process of ZnS, and ZnS hardly affected the formation of CaFe_2_O_4_ at lower temperatures. Therefore, with the increase of Zn content, the LAT of iron ore containing ZnFe_2_O_4_ increased the most, while that of iron ore containing ZnS had no obvious change.

### 3.2. The Effects of Different Forms of Zn on Liquid Phase Fluidity

**Figure 9:**
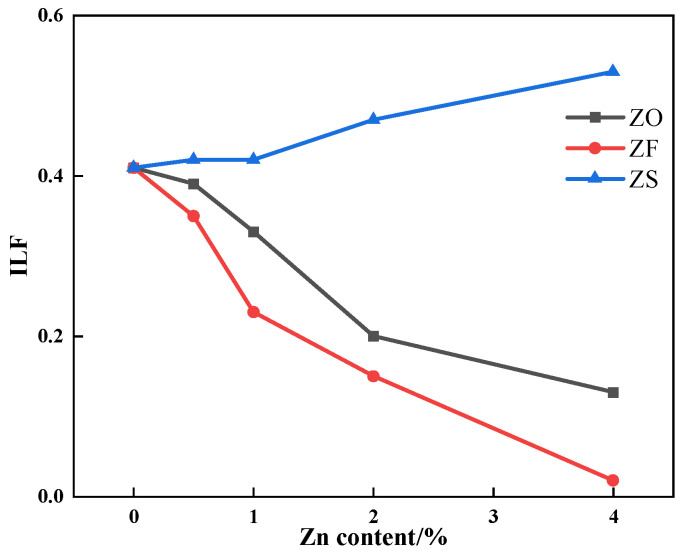
The effects of different forms of Zn on the ILF of iron ore.

The results of the liquid phase fluidity experiment of iron ore containing different contents and forms of Zn are shown in Figure 9. It can be seen from Figure 9 that with increasing the Zn content, the index of liquid phase fluidity (ILF) of iron ore containing ZnS increased while that of iron ore containing ZnO and ZnFe_2_O_4_ decreased. The effect of ZnFe_2_O_4_ on ILF was greater than ZnO when the Zn content was the same.

**Figure 10:**
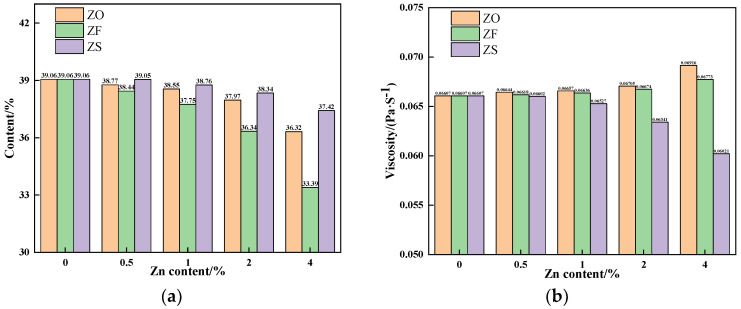
The properties of the equilibrium liquid phase at 1280 °C of experimental samples containing different contents and forms of Zn: (**a**) Contents; (**b**) Viscosities.

The properties of the sintering equilibrium liquid phase of experimental samples containing different contents and forms of Zn are shown in Table 3 and Figure 10. As can be seen from Table 3 and Figure 10a, with the Zn content increased, the Zn^2+^ content in the liquid phase increased while the liquid phase content decreased, and the liquid phase content of the experimental sample containing ZnFe_2_O_4_ had a significant decrease. As shown in Figure 10b, with the increase of Zn content, the liquid phase viscosity of experimental samples containing ZnO and ZnFe_2_O_4_ increased while that of the experimental sample containing ZnS decreased. Research showed that Fe^2+^ broke the chain of polymer macromolecules such as silico-oxygen complex anion groups, destroying the Si-O network structure in the silicate liquid phase, so the viscosity of the liquid phase decreased [25]. As can be seen from Table 3, with the increase of Zn content, Fe^2+^ content in the liquid phase of experimental samples containing ZnO and ZnFe_2_O_4_ decreased, while that of the experimental sample containing ZnS increased. Therefore, under the combined effect of liquid content and viscosity, as the Zn content increased, the ILF of iron ore containing ZnS increased, while that of iron ore containing ZnO and ZnFe_2_O_4_ decreased.

### 3.3. The Effects of Different Forms of Zn on SFCA Formation Characteristics

**Figure 11:**
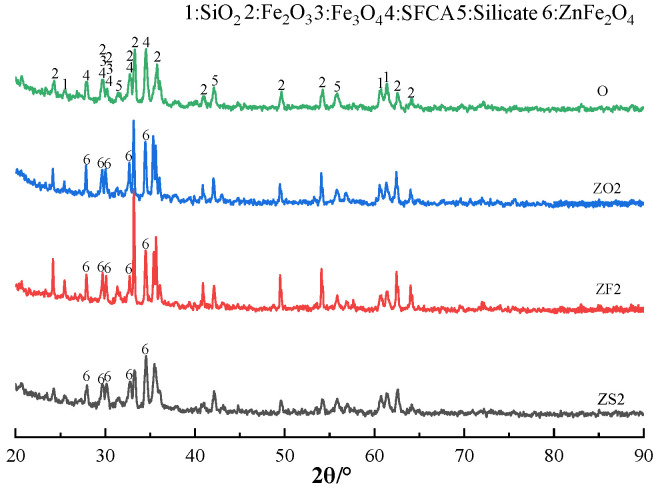
The XRD patterns of sinter after sintering iron ore containing 2% Zn.

The XRD patterns of sinter after sintering iron ore containing 2% Zn are shown in Figure 11. It can be seen from Figure 11 that Zn mainly existed in the form of ZnFe_2_O_4_ in the Zn-containing sinter. Moreover, the diffraction peaks of ZnFe_2_O_4_ and Fe_3_O_4_ coincided perfectly because both of them were spinel minerals.

**Figure 12:**
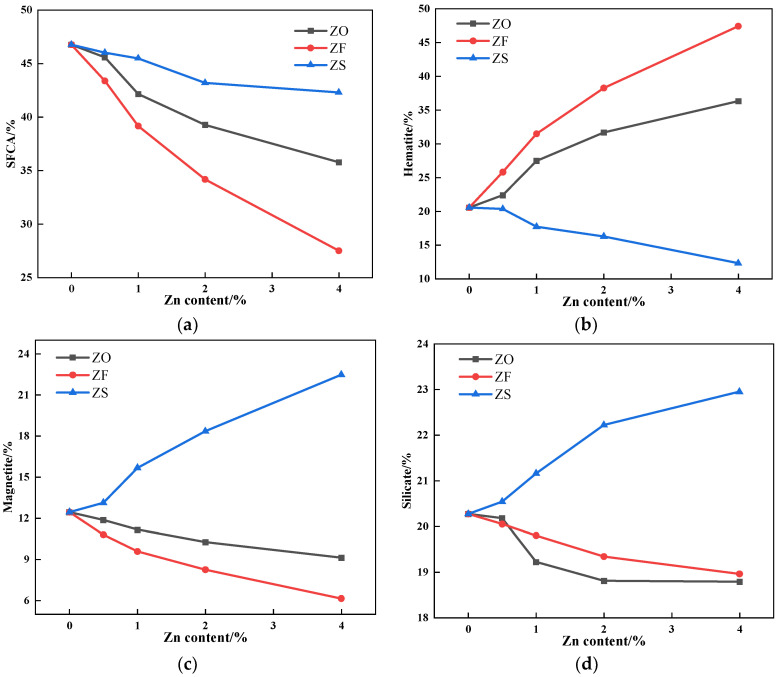
The variation of mineral phase content of sinter after sintering iron ore containing different contents and forms of Zn: (**a**) SFCA; (**b**) Hematite; (**c**) Magnetite; (**d**) Silicate.

The relative area percentage of the mineral phase in the sinter was obtained by Image J 1.54h software, as shown in Figure 12. It can be seen from Figure 12 that with the increase of ZnO and ZnFe_2_O_4_ contents, the contents of silico-ferrite of calcium and aluminum (SFCA), magnetite and silicate decreased, while the content of hematite increased; with the increase of ZnS content, the contents of SFCA and hematite decreased, while that of magnetite and silicate increased.

ZnO and ZnFe_2_O_4_ had a solid-phase reaction with Fe_2_O_3_ and CaO, respectively, during sintering, which inhibited the formation of calcium ferrite, a reactant in the SFCA formation process, and ZnFe_2_O_4_ had a stronger inhibition effect. In addition, the viscosity of the liquid phase increased because of the decrease of Fe^2+^ content in the liquid phase, so the permeability of the liquid phase to the surrounding adhesion layer was weakened, which also inhibited the formation of SFCA. Therefore, the content of SFCA in sinter after sintering iron ore containing ZnFe_2_O_4_ decreased greatly. Although ZnS promoted the decomposition of Fe_2_O_3_ and inhibited the formation of SFCA, there was more Fe^2+^ content in the liquid phase formed by iron ore containing ZnS during sintering. The penetration range of the liquid phase with stronger fluidity was wider than that of the surrounding adhesion layer, which promoted the formation of SFCA. Therefore, the content of SFCA in the sinter after the sintering of iron ore containing ZnS decreased slightly.

### 3.4. The Effects of Different Forms of Zn on Bonding Phase Strength

#### 3.4.1. The Distribution Characteristics of Zn and Microhardness

**Figure 13:**
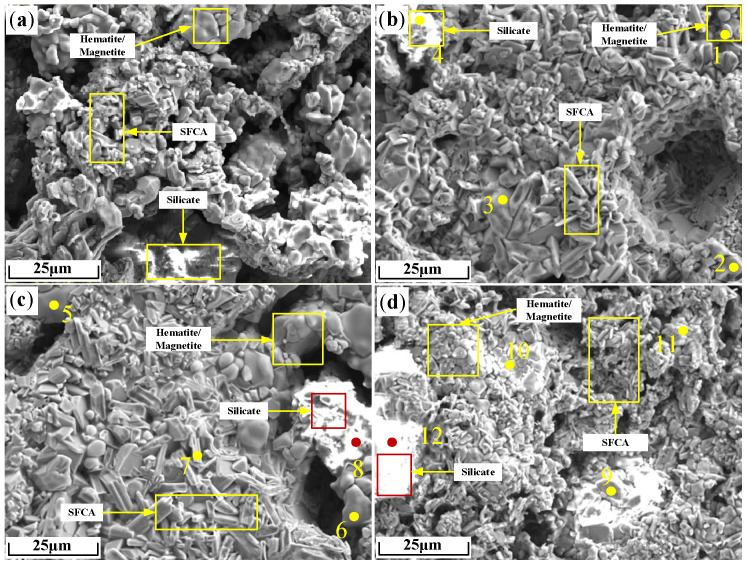
Microstructure of sinter after sintering iron ore containing 2% Zn: (**a**) O; (**b**) ZO2; (**c**) ZF2; (**d**) ZS2.

The microstructure of the sinter after sintering iron ore containing 2% Zn is shown in Figure 13, and the EDS analysis results of points in Figure 13 are shown in Table 4. It can be seen from Table 4 that Zn was mainly distributed in hematite or silicate.

During sintering, ZnO reacted with Fe_2_O_3_ to form ZnFe_2_O_4_, and then, ZnFe_2_O_4_ reacted with CaO to form Ca_2_Fe_2_O_5_ and ZnO; however, the content of CaO in the experimental sample was much lower than that of Fe_2_O_3_. Moreover, SiO_2_ reacted with CaO to form Ca_2_SiO_4_, which restrained the formation of Zn_2_SiO_4_. Therefore, most of Zn in the sinter eventually existed in the hematite in the form of ZnFe_2_O_4_. ZnS began to be oxidized by O_2_ generated by the decomposition of Fe_2_O_3_ at about 1100 °C; CaO was almost exhausted at this temperature. As shown in Figure 11, there was still residual SiO_2_ in the sinter, and the ΔG of the Zn_2_SiO_4_ formation reaction was lower than that of the ZnFe_2_O_4_ formation reaction. Therefore, part of Zn existed in the form of Zn_2_SiO_4_ in the sinter after sintering iron ore containing ZnS. Zn in SFCA and magnetite existed in the form of a solid solution. Spinel polysomes were commonly contained in SFCA mineral structures, which meant that both SFCA and magnetite contained spinel structures [26]. Compared with the Mg^2+^ radius (0.65 Å), the Zn^2+^ radius (0.74 Å) was closer to the Fe^2+^ radius (0.76 Å); hence, Fe^2+^ in SFCA and magnetite was mainly substituted by Zn^2+^.

**Figure 14:**
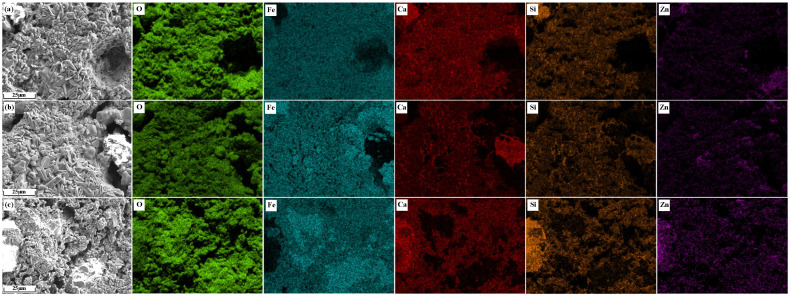
Element distribution of Zn-bearing sinter: (**a**) ZO2; (**b**) ZF2; (**c**) ZS2.

The element distribution of Zn-bearing sinter in Figure 13 is shown in Figure 14. It can be seen from Figure 14a,b that Zn in sinter ZO2 and ZF2 was concentrated in hematite and less distributed in SFCA. Figure 14c shows that Zn in the sinter ZS2 was concentrated in the silicate and overlapped better with the Si element, uniformly distributed in iron-bearing minerals.

To further reveal the mechanism of Zn deviation distribution in SFCA and magnetite, based on first-principles calculations, the adsorption energies of Zn on SFCA and magnetite crystal surfaces were calculated by the Vienna ab initio simulation package (VASP 6.3.2) [27]. The generalized gradient approximation by Perdew–Burke–Ernzerhof (PBE) was employed to describe the exchange–correlation functional [28]. The ion–electron interaction was estimated by the projector-augmented wave model [29]. The energy cutoff for the plane wave expansion was set to 450 eV. A 3 × 3 × 3 Monkhorst Pack k-point setup was used for geometry optimization. The energy convergence for terminating the electronic self-consistent field was 10^−6^ eV/atom, and the force convergence on each atom for geometric optimization was 10^−2^ eV/Å.

The atomic site and cell structure of the 15 × 15 × 15 Å cell with Zn atom in the central location were optimized by the VASP program under the above parameter setting, and the energy of the Zn atom was finally obtained to be −0.2647 eV.

**Figure 15:**
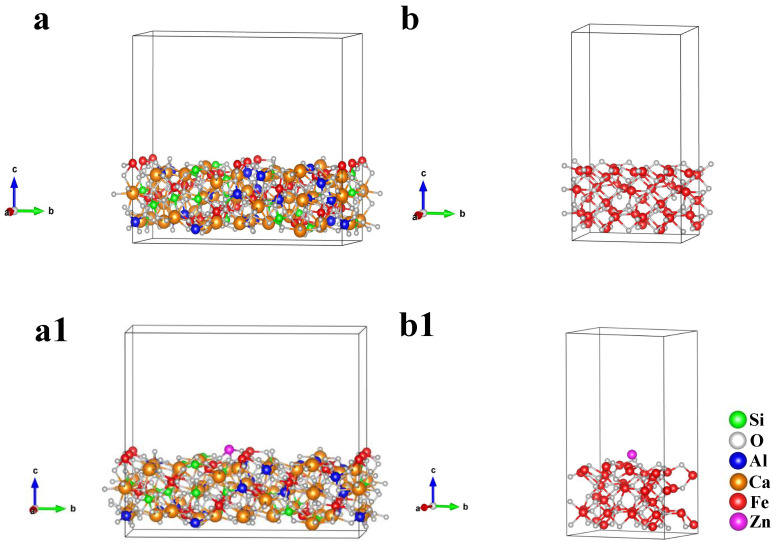
Crystal surface adsorption models of (**a**,**a1**) SFCA (420) and (**b**,**b1**) Fe_3_O_4_ (220).

The cell parameters calculated after optimizing the crystal structure of SFCA and magnetite are shown in Table 5; the deviations between calculation values and experience values of cell parameters are lower than 1%, belonging to a reasonable range [30]. According to the corresponding strongest diffraction peaks of SFCA and magnetite in Figure 11, SFCA (420) and Fe_3_O_4_ (220) crystalline planes were selected to construct the crystal surface adsorption models, as shown in Figure 15.

**Figure 16:**
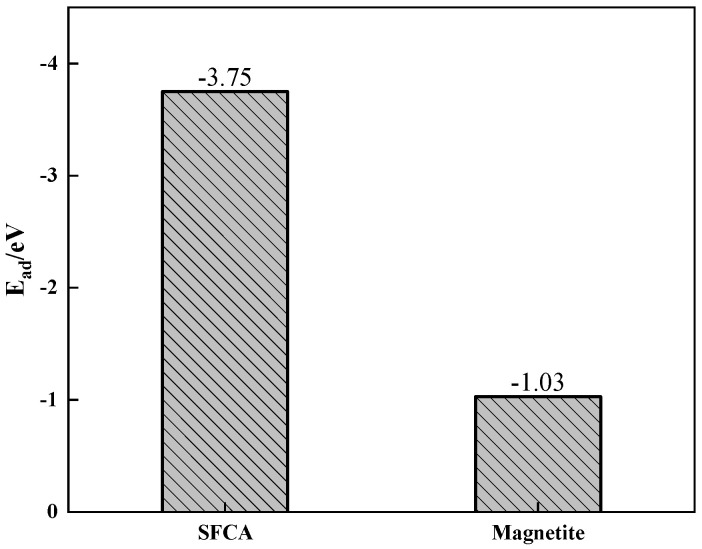
Adsorption energies of Zn on the SFCA and magnetite crystal surfaces.

The adsorption energies of Zn on the SFCA and magnetite crystal surfaces are shown in Figure 16. Figure 16 shows that the SFCA crystal released more energy during the adsorption of Zn, which meant that the SFCA crystal was more stable after the adsorption of Zn. Therefore, more Zn-containing solid solutions were formed in SFCA.

The results of microhardness detection are shown in Table 6. Table 6 shows that with the increase of Zn content, the microhardness of silicate had no obvious change; among the iron-bearing minerals, the microhardness of hematite decreased slightly, while that of SFCA in sinter after sintering iron ore containing ZnO and ZnFe_2_O_4_ decreased significantly.

Combined with the data in Table 4 and Table 6, it can be deduced that the microhardness of the mineral phase was inversely proportional to the amount of Zn-containing solid solution contained in the mineral phase. Therefore, the micromechanical properties of SFCA and magnetite deteriorated because of the formation of Zn-containing solid solution. In addition, the formation of ZnFe_2_O_4_ broke the continuity of the Fe_2_O_3_ crystal bond, resulting in a decrease in the microhardness of hematite.

#### 3.4.2. Compression Strength

**Figure 17:**
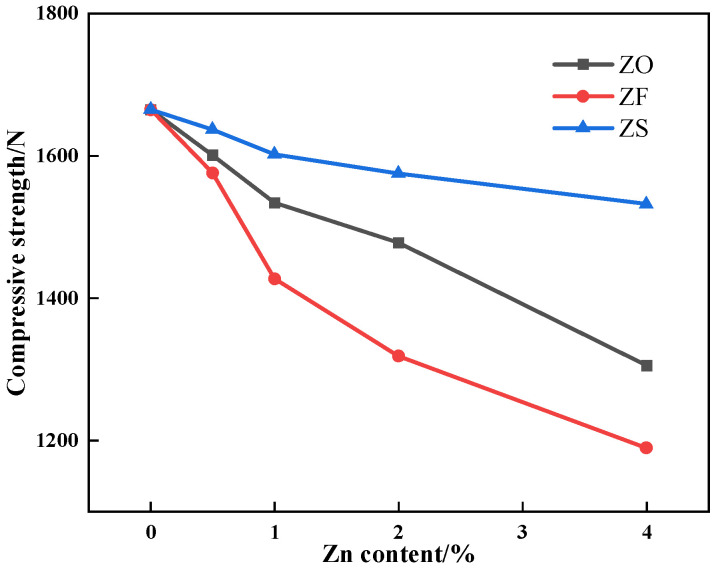
The effects of different forms of Zn on the compressive strength of sinter.

The compressive strength of the sinter after sintering iron ore containing different contents and forms of Zn is shown in Figure 17. Figure 17 shows that the compressive strength of the sinter decreased with the Zn content increased, and the compressive strength of the sinter after sintering iron ore containing ZnFe_2_O_4_ decreased more sharply.

As the microhardness of iron-bearing minerals in the Zn-bearing sinter decreased, the compressive strength of the sinter decreased accordingly. However, it can be seen from Table 6 that ZnO and ZnFe_2_O_4_ had an approximate influence on mineral microhardness, which was inconsistent with the results in the compressive strength experiment. Meanwhile, the effect of Zn on SFCA formation was consistent with that of Zn on the compressive strength of the sinter. Therefore, Zn affected the bonding phase strength by the formation of a solid solution and decreasing the SFCA content.

## 4. Conclusions

In this paper, the micro-sintering method was adopted to measure the sintering basic characteristics of iron ore, and the influence mechanism of ZnO, ZnFe_2_O_4_, and ZnS on sintering basic characteristics of iron ore was determined by first-principles calculations and thermodynamic analysis. The conclusions are as follows:

1. After the addition of ZnO and ZnFe_2_O_4_, the LAT of iron ore increased while the ILF of iron ore decreased. The addition of ZnFe_2_O_4_ consumed limited CaO, so the LAT and ILF of iron ore containing ZnFe_2_O_4_ had a greater variation. As the ZnS content increased, the LAT of iron ore had no obvious change, while the ILF of iron ore increased;
2. The addition of ZnO and ZnFe_2_O_4_ resulted in the formation of ZnFe_2_O_4_ and Ca_2_Fe_2_O_5_, respectively; the content of reactants that participated in the formation of SFCA decreased. Hence, the presence of ZnO and ZnFe_2_O_4_ had a serious impact on SFCA formation during sintering. The addition of ZnS promoted the decomposition of Fe_2_O_3_; however, the process was limited by temperature. Hence, the presence of ZnS had a slight influence on SFCA formation during sintering;
3. The Zn is mainly concentrated in hematite or silicate, less distributed in SFCA and magnetite in the form of a solid solution. Among SFCA and magnetite, the adsorption energy of Zn on the SFCA crystal surface was lower, so the SFCA bonding phase contained more Zn-containing solid solutions, which decreased the microhardness of the mineral phase. The decrease in microhardness and content of the SFCA bonding phase decreased the bonding phase strength;
4. The iron ore with high Zn content presented poor sintering basic characteristics during the sintering process. Hence, both removing the Zn element in dust and ore blending optimization based on sintering basic characteristics are not only good approaches to recovering metallurgical dust and producing high-quality sinter but are also future research directions.

## Figures and Tables

**Table 1:** Chemical Composition of Iron Ore (%).

TFe	CaO	SiO_2_	Al_2_O_3_	MgO	ZnO
61.28	0.17	5.27	2.69	0.26	≤0.01

**Table 2:** Chemical Composition of Iron Ore with Different Contents and Forms of Zn (%).

Samples	TFe	CaO	SiO_2_	Al_2_O_3_	MgO	ZnO	ZnFe_2_O_4_	ZnS
O	61.28	0.17	5.27	2.69	0.26	--	--	--
ZO0.5	60.89	0.17	5.24	2.67	0.26	0.63	--	--
ZO1	60.51	0.17	5.20	2.66	0.26	1.25	--	--
ZO2	59.75	0.17	5.14	2.62	0.25	2.49	--	--
ZO4	58.23	0.16	5.01	2.56	0.25	4.98	--	--
ZF0.5	60.15	0.17	5.17	2.64	0.26	--	1.85	--
ZF1	59.01	0.16	5.08	2.59	0.25	--	3.70	--
ZF2	56.76	0.16	4.88	2.49	0.24	--	7.38	--
ZF4	52.24	0.14	4.49	2.29	0.22	--	14.76	--
ZS0.5	60.82	0.17	5.23	2.67	0.26	--	--	0.75
ZS1	60.37	0.17	5.19	2.65	0.26	--	--	1.49
ZS2	59.45	0.16	5.11	2.61	0.25	--	--	2.98
ZS4	57.63	0.16	4.96	2.53	0.24	--	--	5.96

**Table 3:** The Compositions of Equilibrium Liquid Phase at 1280 °C of Experimental Samples Containing Different Contents and Forms of Zn (%).

Samples	Fe_2_O_3_	Al_2_O_3_	SiO_2_	CaO	FeO	MgO	ZnO
O	57.839	4.085	11.064	22.152	4.730	0.131	0.000
ZO0.5	57.692	4.067	11.106	22.213	4.510	0.088	0.323
ZO1	57.652	4.054	11.106	22.236	4.351	0.068	0.531
ZO2	57.509	4.003	11.167	22.334	4.134	0.047	0.806
ZO4	56.676	3.912	11.426	22.858	3.887	0.029	1.211
ZF0.5	57.768	4.051	11.071	22.187	4.513	0.089	0.321
ZF1	57.682	3.993	11.102	22.252	4.371	0.068	0.532
ZF2	57.532	3.899	11.170	22.371	4.173	0.045	0.810
ZF4	57.103	3.677	11.364	22.719	3.953	0.025	1.158
ZS0.5	57.714	4.052	11.046	22.124	4.740	0.069	0.255
ZS1	57.535	4.020	11.071	22.145	4.812	0.047	0.370
ZS2	53.829	4.015	12.122	24.255	5.084	0.038	0.657
ZS4	51.132	3.992	12.379	26.134	5.476	0.021	0.866

**Table 4:** EDS Analysis Results (at%).

Points	Fe	Ca	Si	Al	Mg	O	Zn	Minerals
1	47.39	1.40	0.12	0.64	0.15	50.04	0.26	magnetite
2	38.95	0.23	0.08	0.42	0.16	59.01	1.15	hematite
3	24.17	6.46	3.02	2.09	0.22	63.64	0.40	SFCA
4	4.41	14.82	9.59	8.04	1.02	61.99	0.13	silicate
5	48.80	0.38	0.16	0.50	0.11	49.90	0.15	magnetite
6	36.26	0.79	0.10	0.57	0.21	60.50	1.57	hematite
7	26.80	8.55	4.25	2.03	0.13	57.82	0.42	SFCA
8	6.93	23.04	12.78	0.34	0.16	56.66	0.09	silicate
9	46.19	0.24	0.16	0.42	0.14	52.77	0.08	magnetite
10	39.07	0.21	0.07	0.41	0.02	59.66	0.56	hematite
11	23.43	6.90	3.69	2.09	0.06	63.71	0.12	SFCA
12	2.30	18.42	12.94	5.32	0.09	59.64	1.29	silicate

**Table 5:** Optimized Cell Parameters of SFCA and Magnetite Crystals (Å).

Crystals		a	b	c
SFCA	Calc	10.080	10.660	9.110
	Exp	10.050	10.558	9.069
	Error	0.30%	0.96%	0.45%
Fe_3_O_4_	Calc	8.399	8.399	8.399
	Exp	8.389	8.389	8.389
	Error	0.12%	0.12%	0.12%

**Table 6:** Microhardness values of mineral phases in sinter after sintering iron ore containing different contents and forms of Zn (HV).

Samples	Silicate	SFCA	Hematite	Magnetite
O	404.3	891.4	1056.5	775.2
ZO0.5	409.5	862.2	1041.3	768.6
ZO1	411.2	815.4	1033.2	762.5
ZO2	401.3	723.1	1035.6	743.1
ZO4	406.3	652.4	1029.7	730.2
ZF0.5	383.2	877.4	1047.4	760.3
ZF1	401.3	832.6	1042.3	755.6
ZF2	397.5	756.7	1039.5	740.9
ZF4	407.4	676.5	1040.3	720.8
ZS0.5	417.4	867.2	1054.1	770.7
ZS1	390.6	857.4	1051.7	767.5
ZS2	410.3	830.5	1052.8	764.2
ZS4	402.7	812.2	1040.9	757.4

## Data Availability

The original contributions presented in the study are included in the article; further inquiries can be directed to the corresponding author.
